# Isolation and Screening of Hydrogen-Oxidizing Bacteria from Mangrove Sediments for Efficient Single-Cell Protein Production Using CO_2_

**DOI:** 10.3390/microorganisms14020346

**Published:** 2026-02-02

**Authors:** Xiaxing Cao, Liang Cui, Shuai Sun, Tingzhao Li, Yong Wang, Shasha Wang, Rongfeng Hong, Pufan Xu, Xuewen Gao, Lijing Jiang, Zongze Shao

**Affiliations:** 1 Key Laboratory of Marine Genetic Resources, Third Institute of Oceanography, Ministry of Natural Resources of PR China, Xiamen 361005, China; caoxiaxing@tio.org.cn (X.C.); cuiliang@tio.org.cn (L.C.); wangyong@tio.org.cn (Y.W.); wangshasha@tio.org.cn (S.W.); 13808528741@163.com (R.H.); xupufan2002@163.com (P.X.); 22320241151493@stu.xmu.edu.cn (X.G.); 2 Amway (Shanghai) Innovation & Science Co., Ltd., Shanghai 201203, China; shuai.sun@amway.com (S.S.); teric.li@amway.com (T.L.); 3 Amway (China) Botanical R&D Center, Wuxi 214115, China; 4 School of Marine Biology, Xiamen Ocean Vocational College, Xiamen 361012, China

**Keywords:** hydrogen-oxidizing bacterium (HOB), single-cell protein (SCP), reverse tricarboxylic acid (rTCA), chemolithoautotrophic, mangrove sediments

## Abstract

The escalating global demand for large-scale, cost-effective, and sustainable high-quality protein has positioned single-cell protein (SCP) production from one-carbon (C1) gases as a highly promising solution. In this study, eight chemolithoautotrophic hydrogen-oxidizing bacteria (HOB) were isolated from mangrove sediments. Based on the 16S rRNA gene sequence analysis, they belonged to genera *Sulfurimonas*, *Sulfurovum*, *Thiomicrolovo*, and *Marinobacterium.* Among these, *Thiomicrolovo* sp. ZZH C-3 was identified as the most promising candidate for SCP production based on the highest biomass and protein content, and was selected for further characterization. Strain ZZH C-3 is a Gram-negative, short rod-shaped bacterium with multiple flagella. It can grow chemolithoautotrophically by using molecular hydrogen as an energy source and molecular oxygen as an electron acceptor. Genomic analysis further confirmed that ZZH C-3 harbors a complete reverse tricarboxylic acid (rTCA) cycle gene set for carbon fixation, and diverse hydrogenases (Group I, II, IV) for hydrogen oxidation. Subsequently, its cultivation conditions and medium composition for SCP production were systematically optimized using single-factor experiments and response surface methodology (RSM). Results showed that the optimal growth conditions were 28 °C, pH 7.0, and with 1 g/L (NH_4_)_2_SO_4_ as the nitrogen source, 5–10% oxygen concentration, 9.70 mg/L FeSO_4_·7H_2_O, 0.17 g/L CaCl_2_·2H_2_O, and 1.90 mg/L MnSO_4_·H_2_O. Under the optimized conditions, strain ZZH C-3 achieved a maximum specific growth rate of 0.46 h^−1^. After 28 h of cultivation, the optical density at 600 nm (OD_600_) reached 0.94, corresponding to a biomass concentration of 0.60 g/L, and the protein content ranked at 73.56%. The biomass yield on hydrogen (Y_H2_) was approximately 3.01 g/g H_2_, with an average H_2_-to-CO_2_ consumption molar ratio of about 3.78. Compared to the model HOB *Cupriavidus necator*, strain ZZH C-3 exhibited a lower H_2_/CO_2_ consumption ratio, superior substrate conversion efficiency, and high protein content. Overall, this study not only validated the potential of mangrove HOB for SCP production but also offers new insights for future metabolic engineering strategies designed to enhance CO_2_-to-biomass conversion efficiency.

## 1. Introduction

Protein is one of the seven essential nutrients for the human body and is crucial for maintaining human nutrition and health. The global population is projected to reach 9.7 billion by 2050 [1]. To meet current demand for animal-derived protein, annual global production of 1.25 billion tons of meat and dairy products is necessary, placing immense pressure on existing protein production systems [2]. However, traditional animal and plant protein production is characterized by low resource conversion efficiency and exerts significant environmental impacts, including substantial greenhouse gas emissions, extensive land use, and water eutrophication [3]. Carbon dioxide (CO_2_) is the most influential greenhouse gas, accounting for 49% of the effect and is simultaneously the planet’s most abundant carbon resource [4]. Therefore, the development of alternative protein production technologies capable of directly utilizing CO_2_, achieving high conversion efficiency, and operating in an environmentally friendly manner has become a strategic necessity to tackle the intertwined crises of global food security and climate change.

Single Cell Protein (SCP), also referred to as Microbial Protein (MP), is widely regarded as a pivotal solution to the global protein shortage, owing to its high protein content (30–80%), rich profile of essential amino acids, short production cycle, and low environmental impact [5]. A central challenge for its industrialization lies in securing low-cost, sustainable carbon sources while minimizing the overall carbon footprint of production [6]. The metabolic diversity of bacteria provides a wide range of substrate options for SCP. Among these, One-Carbon Gas Protein (C1GP) produced using C1 compounds like CO_2_, CH_4_, and methanol as substrates stands out for its exceptional economic and environmental potential [7,8]. Consequently, within the dual context of global food security and the urgency of carbon emission reduction, the development of C1GP technology presents vast prospects. Among microorganisms for C1GP production, HOB are a prominent group of chemolithoautotrophs. They utilize hydrogen (H_2_) as an electron donor, oxygen (O_2_) as an electron acceptor, and CO_2_ as their sole carbon source for growth and have shown significant potential in CO_2_ capture and SCP production [9,10]. HOB are categorized into aerobic and anaerobic (acetogenic) types. Current industrial SCP production relies on aerobic HOB like *Cupriavidus necator* and *Hydrogenophaga pseudoflava*, which primarily employ the Calvin Benson Bassham (CBB) cycle for CO_2_ fixation [11]. However, this pathway, faces inherent energy efficiency constraints [12]. First, its key enzyme, Rubisco, is susceptible to oxygen inhibition, leading to energy and carbon wasting oxygenation reactions. Second, the fixation of one CO_2_ molecule consumes 3 ATP and 2 NADPH, representing a substantial energy cost [13]. Although the Wood-Ljungdahl (WL) pathway for carbon fixation used by anaerobic acetogens is highly energy-efficient, its metabolic flux is typically directed toward byproducts such as acetate rather than cellular biomass [14,15]. In addition, other carbon fixation pathways, such as the rTCA cycle, although they possess a clear theoretical advantage in energy efficiency, systematic experimental data on their actual performance in SCP production remain scarce. Therefore, screening for chemolithoautotrophic HOB with high energy conversion efficiency and high protein synthesis capability is crucial for advancing HOB-based SCP production technology.

Coastal sedimentary environments, such as mangroves, are characterized as typically “blue carbon” ecosystems. The dynamic redox gradients within these environments support a rich diversity of chemolithoautotrophic microorganisms, which are fueled by different energy sources such as hydrogen [16]. Therefore, the mangrove ecosystem can serve as a valuable natural resource bank that can be utilized to isolate strains capable of efficiently producing SCP using CO_2_ and H_2_.

In this study, we isolated chemolithoautotrophic HOB from mangrove sediments from Zhangjiang Estuary Mangrove Reserve in Zhangzhou, China. Among isolated HOB, *Thiomicrolovo* sp. ZZH C-3 holds significant potential for industrial applications by physiological and genomic analysis, along with protein content assessments. Its fermentation conditions were furthermore systematically optimized for SCP production. Finally, we compared strain ZZH C-3 with the widely utilized model organism *Cupriavidus necator* in terms of growth rate, protein content, hydrogen conversion capability, and other relevant factors. The results highlight the significant potential of marine HOB strain ZZH C-3 for future large-scale production.

## 2. Materials and Methods

### 2.1. Sample Collection and Strain Enrichment

Mangrove sediment samples were collected from the Zhangjiangkou Mangrove Reserve (117.9° E, 24.4° N) in Zhangzhou, China. Enrichment cultures were established in MMJH medium [17]. The medium (10 mL) was dispensed into 50 mL serum vials and sealed with butyl rubber stoppers under a gas mixture of 72% H_2_/18% CO_2_/10% O_2_ (200 kPa), providing H_2_ as the energy source, CO_2_ as the carbon source, and O_2_ as the terminal electron acceptor. The serum vials were incubated at 30 °C with shaking at 150 rpm. After 3–7 days, microbial growth was evidenced by visible turbidity. Active cultures were serially transferred into fresh medium to enrich for HOB.

### 2.2. Isolation and Identification of Strains

Pure cultures were obtained using the dilution-to-extinction method. The purity was confirmed by microscopic examination and 16S rRNA gene sequencing. For morphological observation, cells were harvested after 16 h of growth in MMJH medium. Cell morphology was observed using an optical microscope (Eclipse 80i, Nikon, Tokyo, Japan) and a transmission electron microscope TEM (Model JEM-1230; JEOL, Tokyo, Japan). Gram staining was performed using a commercial kit (Qingdao Haibo, Qingdao, China). Cellular fatty acids were extracted following a modified Bligh-Dyer method [18].

Molecular identification was carried out by amplifying the 16S rRNA gene using universal primers 27F (5′-AGATTTGATCCTCGTCAG-3′) and 1492R (5′-GGTACCTTTTTACGACT-3′) [19]. The resulting sequences were aligned and analyzed using the EzBioCloud server (https://www.ezbiocloud.net/, accessed on 20 May 2024) and the NCBI database. Alignment with a representative set of related 16S rRNA gene sequences was carried out with the CLUSTAL_W program implemented in the phylogenetic analysis package MEGA version 6.0 [20]. Phylogenetic trees based on the neighbor-joining [21], maximum-likelihood [22] methods were reconstructed and genetic distances were calculated using the Kimura two-parameter model. Bootstrap values were calculated based on 1000 replications.

### 2.3. Optimization of Cultivation Conditions

A systematic optimization of culture conditions was conducted using a shake-flask fermentation system, focusing on temperature, pH, nitrogen source, oxygen concentration, and inorganic salts. Glycerol stocks stored at −80 °C were first inoculated into MMJH medium and pre-cultured at 30 °C and 150 rpm for 18–24 h to prepare the seed culture. Subsequently, a 10% (*v*/*v*) inoculum of the seed culture was transferred into 150 mL serum vials containing 30 mL of medium. Cultivation was carried out under the same conditions, with biomass, protein content, and protein yield measured at 2–3 h intervals.

#### 2.3.1. Optimization of Initial pH and Temperature

The initial pH of the medium was adjusted to 5.0, 6.0, 7.0, and 8.0 using appropriate buffer systems. Incubation temperatures tested were 25, 28, 30, 32, and 37 °C. The optical density at 600 nm (OD_600_) was measured every 2–3 h to plot growth curves and determine the optimal pH and temperature.

#### 2.3.2. Optimization of Nitrogen Source

Four different nitrogen sources ammonium sulfate, ammonium chloride, sodium nitrate, and urea were supplemented into the medium at an equivalent nitrogen concentrations of 100 mg N/L. Upon determining the optimal nitrogen source, the impact of different concentrations (0.5–4 g/L) on the growth of *Thiomicrolovo* sp. ZZH C-3 was further assessed.

#### 2.3.3. Optimization of Oxygen Concentration

The optimal oxygen concentration for growth was determined by varying the O_2_ content in the headspace gas mixture. While maintaining a constant H_2_:CO_2_ ratio (8:2, *v*/*v*), the balance of the gas phase was composed of O_2_ and N_2_ at different ratios to achieve final O_2_ concentrations of 1%, 5%, 10%, 20%, 30%, and 40% (*v*/*v*). Samples were taken every 2–3 h for OD_600_ measurement, and growth curves were plotted to determine the optimal oxygen concentration.

#### 2.3.4. Effect of Auxiliary Energy Sources

To evaluate the effect of auxiliary energy sources, 10 mM sodium thiosulfate (Na_2_S_2_O_3_) or sodium tetrathionate (Na_2_S_4_O_6_) was added to the culture medium with H_2_ as the primary energy source. A control experiment with H_2_ as the sole energy source (no sulfur compounds added) was conducted in parallel. Growth was assessed by monitoring OD_600_ at 2–3 h intervals.

#### 2.3.5. Optimization of the Medium Composition by RSM

A Plackett-Burman (PB) experimental design was first employed to screen 11 medium components for their significant effects on the biomass accumulation of *Thiomicrolovo* sp. ZZH C-3. Each factor was tested at two levels: low (−1) and high (+1) (Table S2). This was followed by the method of steepest ascent to approximate the optimal region and determine the center point for subsequent Box–Behnken Design (BBD) (Table S4). A BBD was then implemented with FeSO_4_·7H_2_O (A), CaCl_2_·2H_2_O (B), and MnSO_4_·H_2_O (C) as independent variables and OD_600_ as the response. The experimental design and data analysis were performed using Design-Expert software (Version 13) (Table S5).

### 2.4. Analytical Methods

#### 2.4.1. Genome Sequencing and Analysis

Genomic DNA was extracted from pure cultures of *Thiomicrolovo* sp. ZZH C-3 during the mid-exponential growth phase using a commercial bacterial DNA extraction kit [23]. Whole-genome sequencing was conducted on the Illumina HiSeq 2000 platform (Meiji Biotechnology Co., Ltd., Shanghai, China). The genomic G+C content was calculated directly from the assembled sequence. Gene prediction and functional annotation were performed using the NCBI Prokaryotic Genome Annotation Pipeline (PGAP) [24], KEGG, and RAST databases [25]. The Average Nucleotide Identity (ANI) was calculated with Ortho-ANI [26], and digital DNA-DNA hybridization (dDDH) values were estimated using the Genome-to-Genome Distance Calculator (GGDC) web service (https://ggdc.dsmz.de/, accessed on 20 June 2024).

#### 2.4.2. Gas Composition Analysis

The composition of the headspace gas was analyzed using a gas chromatograph (Fuli F60, Fuli Instruments, Taizhou, China) equipped with a TDX-01 packed column (3 mm × 2 m) and a thermal conductivity detector (TCD) [27]. Argon served as the carrier gas, and the column temperature was maintained at 120 °C. A sterile gas-tight syringe (Fuli Instruments, Taizhou, China) was used to periodically withdraw 0.3 mL samples from the headspace for the quantification of H_2_ and CO_2_ concentrations.

#### 2.4.3. Product Analysis

##### Biomass Determination

To establish a correlation between optical density and cell dry weight (CDW), *Thiomicrolovo* sp. ZZH C-3 was cultivated in a 1 L serum vials containing 200 mL of MMJH medium under the standard conditions (30 °C, 150 rpm). Aliquots of 400 mL of bacterial suspension, covering an OD_600_ range of 0.1 to 0.8, were centrifuged at 10,000 rpm for 15 min. The cell pellets were washed three times with phosphate-buffered saline (PBS) and then dried in an oven at 65 °C for 48 h until a constant weight was achieved. The dry mass was measured to determine the cell dry weight (CDW). A linear standard curve relating OD_600_ to CDW was generated (Figure S1) and used for all subsequent biomass estimations.

##### Protein Content Analysis

The crude protein content of the biomass, expressed as a percentage of CDW, was determined via elemental nitrogen analysis. Briefly, lyophilized cell pellets were finely ground. A 3–5 mg aliquot of the homogeneous powder was weighed into a tin capsule and analyzed for total nitrogen content using an elemental analyzer (Flash 2000, Thermo Fisher Scientific, Waltham, MA, USA). The crude protein content was calculated by multiplying the measured nitrogen content by the conventional conversion factor of 6.25.

##### Measurement and Composition Analysis of Extracellular Polysaccharide (EPS) Components

EPS were extracted using a modified thermal method [28], and the polysaccharide content was quantified by the phenol-sulfuric acid method. The monosaccharide compositions of the EPS were analyzed by LC-MS [29].

### 2.5. Statistical Analysis

For single-factor optimization experiments, data are presented as the mean ± standard deviation (SD) of at least three independent replicates. Differences between treatment groups were evaluated using one-way analysis of variance (ANOVA), followed by the least significant difference (LSD) post hoc test for multiple comparisons when the ANOVA indicated a significant effect (*p* < 0.05). The PB and BBD experimental designs, along with the corresponding regression analysis and ANOVA for the model, were performed using Design-Expert software (Version 13, Stat-Ease Inc., Minneapolis, MN, USA). All other graphs were generated using Origin Pro 2024 (Origin Lab Corp., Northampton, MA, USA).

## 3. Results

### 3.1. Isolation and Screening of HOB for SCP Production

**Figure 1:**
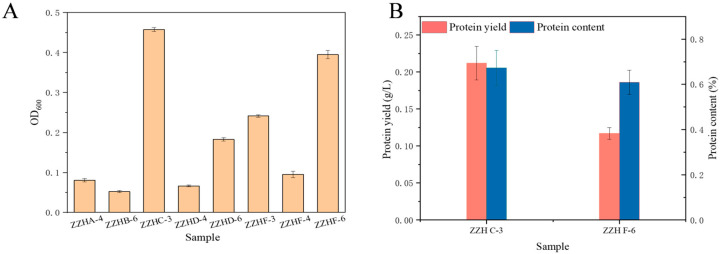
Evaluation of biomass and protein production capacity of isolated HOB strains from mangrove sediments. (**A**) biomass of the 8 screened strains. (**B**) the protein content and protein yield of ZZH C-3 and ZZH F-6.

A total of eight strains were successfully isolated from mangrove sediments. They mainly included *Sulfurimonas*, *Sulfurovum*, *Thiomicrolovo*, and *Marinobacterium* (Table S1). Considering that protein yield is closely related to bacterial biomass, we first conducted an initial assessment of the biomass of these strains during chemolithoautotrophic growth. After 24 h of shake-flask cultivation, four strains (ZZH A-4, ZZH B-6, ZZH D-4, ZZH F-4) exhibited OD_600_ below 0.10, two strains (ZZH D-6, ZZH F-3) have OD_600_ ranging from 0.10–0.30, and two strains (ZZH C-3, ZZH F-6) demonstrated best growth, with OD_600_ reaching of 0.46 and 0.40, respectively (Figure 1A). Subsequently, the protein production capacity was further determined in the two strains. The results revealed that strain ZZH C-3 achieved a protein content of 67.30% and a protein yield of 0.21 g/L, which was higher than those of strain ZZH F-6 (60.91% and 0.12 g/L, respectively) (Figure 1B). Based on its combined advantages in both biomass and protein synthesis capability, ZZH C-3 was selected as the most promising candidate for SCP production and was subjected to further investigation.

### 3.2. Physiological and Phylogenetic Characterizations of Strain ZZH C-3

**Figure 2:**
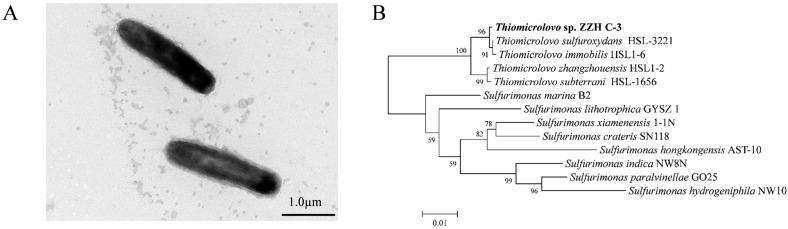
Morphology and phylogenetic analysis of the strain ZZH C-3. (**A**) Transmission electron microscopy image of ZZH C-3; (**B**) Phylogenetic tree based on 16S rRNA gene sequences showing the position of strain ZZH C-3 within the genus *Thiomicrolovo*.

Strain ZZH C-3 is a Gram-negative bacterium with a slightly curved, short-rod morphology. The cells are approximately 1.5–2.0 μm in length and 0.4–0.5 μm in width, and possess multiple fimbriae (Figure 2A). To determine its taxonomic position, the phylogenetic analysis based on 16S rRNA gene was further performed. The result showed this bacterium clustered within the genus *Thiomicrolovo* (Figure 2B). The 16S rRNA sequence of ZZH C-3 showed the highest similarity (99.67%) to *Thiomicrolovo sulfuroxydans* HSL-3221, which was also isolated from a mangrove sediment. The sequence similarity of this bacterium with other species in the genus ranges from 97.94% to 99.48%. Although the threshold for 16S rRNA-based species delineation is generally considered to be around 98.65%, modern bacterial taxonomy also emphasizes dDDH and ANI as criteria for species circumscription, with established thresholds of dDDH < 70% and ANI < 95–96% [30,31]. Whole-genome ANI analysis yielded values of 89.37% against the respective reference strain *T. sulfuroxydans* HSL-3221, which was below the established 95–96% species boundary. Consistently, dDDH value between strains ZZH C-3 and *T. sulfuroxydans* HSL-3221 was 38%, falling beneath the 70% threshold for species demarcation [32]. Collectively, these data suggest that strain ZZH C-3 could represent a novel species within the genus *Thiomicrolovo*.

Analysis of cellular fatty acid composition provided further support for its phylogenetic classification. The major fatty acids in strain ZZH C-3 were C_16:1_ ω7c (33.55%), C_16:0_ (18.44%), and C_18:1_ ω7c (reported as Summed Feature 8, 13.98%) (Table 1). These three components are also dominant in other *Thiomicrolovo* species. Notably, ZZH C-3 displayed a slightly higher proportion of C_14:0_ (12.53%) than most reference strains, it is the C_16:0_ content (18.44%) was markedly lower than that of *T. sulfuroxydans* HSL-3221 (27.74%) and *T. immobilis* HSL1–6 (28.36%). These differences further support the delineation of ZZH C-3 as a novel species.

### 3.3. Genome Analysis of Strain ZZH C-3

Whole-genome sequencing analysis revealed that the genome of strain ZZH C-3 is 2,615,756 bp (2.62 Mb) in size, containing 2631 protein-coding sequences and 57 RNA genes. The genomic DNA G+C content of ZZH C-3 was 57.49 mol%, which is consistent with the known range for the genus *Thiomicrolovo* (57.2–57.5 mol%). For carbon fixation pathway, ZZH C-3 encodes all essential enzymes for a complete rTCA cycle, including ATP dependent citrate lyase (Acl), pyruvate: ferredoxin oxidoreductase (Por), and 2-oxoglutarate: ferredoxin oxidoreductase (Oor). Although Por and Oor are typically oxygen sensitive, restricting the rTCA cycle to microaerophilic or anaerobic lineages such as *Pseudomonadota*, *Campylobacterota*, and *Aquificales*, aerobic operation of the rTCA cycle in *Hydrogenobacter thermophilus* relies on oxygen tolerant, five subunit isoforms of Oor and Por [33,34]. ZZH C-3 exhibits robust growth under 10% oxygen, indicating the presence of an oxygen-tolerant enzyme system. Distinct from the five-subunit aerobic forms found in some oxygen-tolerant bacteria or the two-subunit versions in strict anaerobes, ZZH C-3 and other *Thiomicrolovo* strains (HSL1–2, HSL-1656, HSL-3221, and HSL1–6) possess four-subunit Oor and Por complexes, suggesting a conservation within genus *Thiomicrolovo*, and potentially representing an adaptation to low or fluctuating oxygen regimes (Table 2).

**Figure 3:**
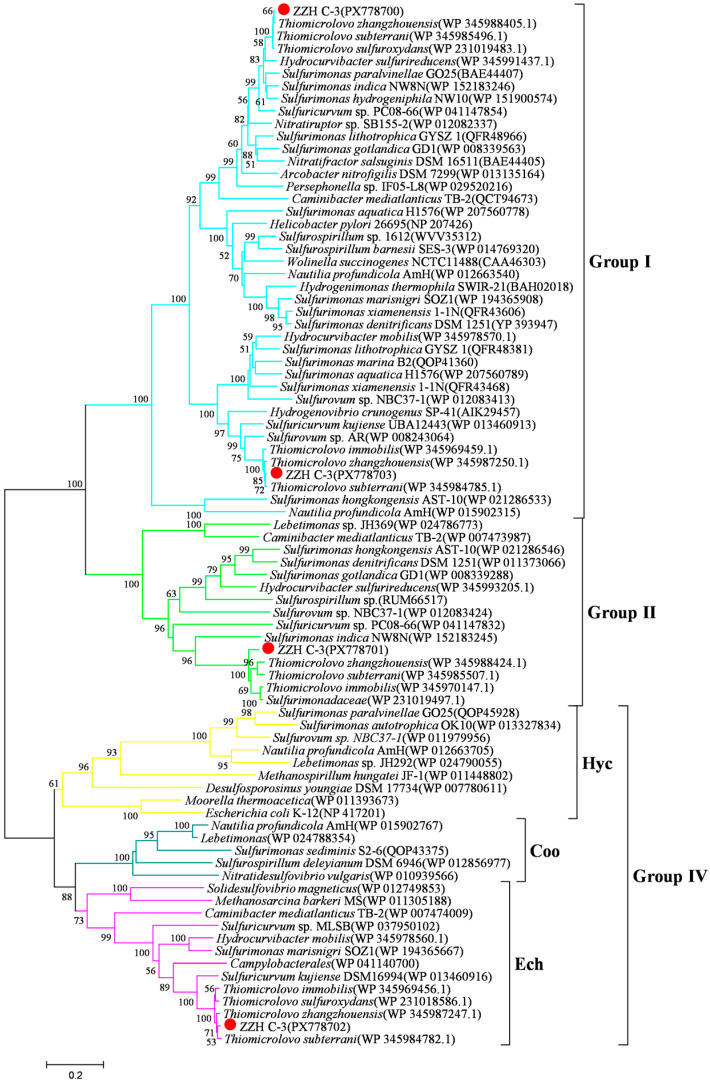
A Maximum-likelihood phylogenetic tree based on large subunit sequences of hydrogenase from *Thiomicrolovo* sp. ZZH C-3 and other representative species within the class Campylobacteria. The solid red circles represent our own isolated strains. Branches representing different hydrogenase groups are different colors.

For hydrogen oxidation, strain ZZH C-3 contains three different types of [Ni-Fe]-hydrogenases (Group I, II, and IV) based on the phylogenetic analysis of hydrogenases, indicating a high metabolic versatility and environmental adaptability with respect to hydrogen utilization (Figure 3). Group I hydrogenase, a membrane-associated [Ni-Fe]-hydrogenase, is directly coupled to respiratory hydrogen oxidation. It transfers electrons derived from hydrogen oxidation to the electron transport chain, thereby driving the reduction in various terminal electron acceptors such as oxygen, nitrate, or elemental sulfur. Strain ZZH C-3 possesses two copies of Group I hydrogenase, which may enable efficient hydrogen oxidation under varying hydrogen concentrations and allow the organism to adapt to fluctuations in hydrogen availability in the in situ environment [35]. Group II hydrogenase differs structurally from Group I, and its small subunit lacks an N-terminal signal peptide, indicating a cytoplasmic localization and no involvement in transmembrane electron transfer. This type of hydrogenase is generally considered to function under low-hydrogen conditions, likely participating in hydrogen sensing or energy conversion at low hydrogen concentrations [36]. The presence of a Group II hydrogenase in ZZH C-3 suggests that the strain can sense and respond to trace levels of hydrogen, which could help maintain metabolic activity under hydrogen-limited conditions. Additionally, ZZH C-3 encodes a Group IV hydrogenase (Ech type). This multi-subunit, membrane-bound hydrogenase acts as an energy-converting hydrogenase that catalyzes proton reduction to molecular hydrogen, thereby dissipating excess reducing equivalents generated during the anaerobic oxidation of low-potential C1 compounds such as carbon monoxide or formate. The Ech hydrogenase couples hydrogen production to ferredoxin reduction and plays a key role in intracellular hydrogen cycling and energy conservation [37]. The presence of this enzyme further expands the metabolic potential of ZZH C-3 under anaerobic or micro-oxic conditions. The complete suite of Group I, II, and IV hydrogenases equips ZZH C-3 with the ability to utilize hydrogen efficiently across a wide range of hydrogen concentrations and redox conditions, thereby enhancing its survival and adaptive capacity in dynamic environments.

In summary, the genome of strain ZZH C-3 is characterized by a complete, oxygen-tolerant rTCA cycle and a versatile suite of hydrogenases. These genomic traits provide a robust genetic foundation for efficient chemolithoautotrophic carbon fixation and biomass synthesis, highlighting its unique metabolic advantages for potential application in aerobic SCP production systems.

### 3.4. Optimization of Cultivation Conditions

To achieve maximum biomass and protein yield of *Thiomicrolovo* sp. ZZH C-3, we systematically optimized key fermentation parameters and medium composition through single-factor and response surface experiments. The targeted parameters included initial pH, temperature, nitrogen source, oxygen, and medium composition.

#### 3.4.1. Optimization of Initial pH and Temperature

**Figure 4:**
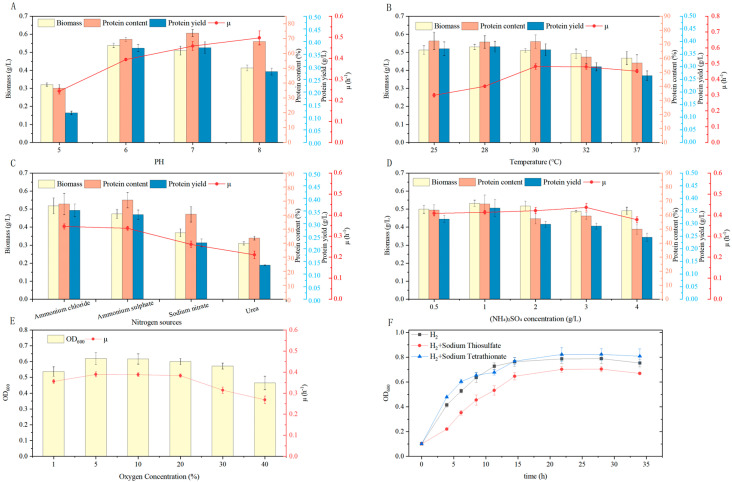
Effects of different culture conditions on biomass, protein content, and protein yield of *Thiomicrolovo* sp. ZZH C-3 (**A**) pH levels; (**B**) temperatures; (**C**) nitrogen source; (**D**) (NH4)_2_SO_4_ concentrations; (**E**) Oxygen Concentration; (**F**) Auxiliary Energy Sources.

Strain ZZH C-3 grew over a pH range of 5.0 to 8.0, but exhibited a clear preference for neutral conditions in terms of both growth and protein synthesis (Figure 4A). The maximum growth rate during the initial exponential phase (0–2 h) increased with pH, which could be consistent with the solubility behavior of CO_2_ [38]. While the highest biomass (0.54 g/L) was achieved at pH 6.0, the maximum protein yield (0.38 g/L) and specific growth rate (0.46 h^−1^) were observed at pH 7.0. Growth was significantly reduced at pH 5.0 (biomass 0.32 g/L) and alkaline conditions (pH 8.0). Previous studies suggest that under pH stress, a greater proportion of energy from hydrogen oxidation is diverted to cellular maintenance [39]. Therefore, considering biomass, protein yield, and growth rate together, pH 7.0 was selected as the optimal culture condition.

For the determination of optimal temperature, results identified ZZH C-3 as a mesophilic bacterium (Figure 4B). Both biomass (0.53 g/L) and protein yield (0.38 g/L) reached their maxima at 28 °C. Although a higher maximum specific growth rate (0.48 h^−1^) was recorded at 30 °C, the final biomass and protein yield (0.51 g/L and 0.37 g/L, respectively) were lower than those at 28 °C. A further increase to 32 °C resulted in reduced growth rates and lower protein content. Additionally, in aerobic fermentation, elevated temperatures reduce gas solubility as described by Henry’s law. It is noteworthy that the solubility of CO_2_ decreases more sharply than that of H_2_ or O_2_, potentially making carbon availability a key growth-limiting factor at higher temperatures [40]. Based on a comprehensive evaluation of final product yield, and energy efficiency, the temperature of 28 °C was selected as the optimal cultivation temperature for subsequent studies.

#### 3.4.2. Optimization of Nitrogen Source

Nitrogen sources are essential precursors for microbial protein synthesis. The effects of different inorganic nitrogen sources on the growth of strain ZZH C-3 were evaluated in this study (Figure 4C). The results demonstrated that among the four tested nitrogen sources, the strain efficiently utilized ammonium salts, achieving relatively high biomass (0.52 g/L and 0.47 g/L) and the highest protein content (67.93% and 70.76%) with ammonium chloride and ammonium sulfate, respectively. In contrast, the biomass of 0.31 g/L and the protein content of 43.61% were lowest with urea as the nitrogen source. Given the higher protein content obtained with ammonium sulfate and to avoid potential growth inhibition from elevated chloride concentrations, ammonium sulfate was selected for optimal nitrogen source for SCP production [41]. We further optimized the concentration of ammonium sulfate concentration on biomass and protein yield (Figure 4D). Both biomass (0.56 g/L) and protein yield (0.38 g/L) reached their maximum values at an ammonium sulfate concentration of 1 g/L. The maximum specific growth rate remained relatively stable across most nitrogen concentrations but decreased markedly at 4 g/L. Therefore, ammonium sulfate at a concentration of 1 g/L was selected as the optimal nitrogen source condition.

#### 3.4.3. Optimization of Oxygen Concentration

As an aerobic HOB, ZZH C-3 uses oxygen as a terminal electron acceptor. However, high oxygen concentrations can inhibit oxygen-sensitive enzymes such as hydrogenases and carbon-fixing enzymes. This study examined the effect of oxygen concentrations ranging from 1% to 40% on its growth (Figure 4E). The results showed that ZZH C-3 exhibited optimal growth under microaerobic conditions at 5% and 10% oxygen, achieving the highest biomass (OD_600_ 0.62) and maximum specific growth rate (0.39 h^−1^). At 1% oxygen, growth was limited by insufficient gas–liquid mass transfer. When oxygen levels increased to 20–40%, growth continued, but final biomass accumulation declined, likely due to energy diversion toward antioxidant stress responses and/or the onset of endogenous respiration [42]. Notably, ZZH C-3 was able to grow even at 40% oxygen (OD_600_ 0.47), indicating robust protective mechanisms for its hydrogenase and carbon fixation systems. This trait is also observed in other SCP-producing strains such as the marine bacterium *Hydrogenovibrio marinus* MH-110, which also tolerates high oxygen levels [43]. From an application perspective, optimal microaerobic conditions (5–10% O_2_) not only satisfy physiological demands but also help minimize aeration costs in industrial fermentation processes. Moreover, reduced oxygen partial pressure indirectly improves the gas–liquid mass transfer efficiency of H_2_ and CO_2_.

#### 3.4.4. Effect of Auxiliary Energy Sources

Although strain ZZH C-3 was isolated as a HOB, members of the class *Campylobacteria* typically exhibit mixotrophic potential. Previous studies suggest that their growth can be enhanced through the synergistic use of multiple electron donors and acceptors [35]. To explore its capacity for utilizing reduced sulfur compounds as an auxiliary energy source, we supplemented the H_2_-based medium with either 10 mM sodium thiosulfate (Na_2_S_2_O_3_) or sodium tetrathionate (Na_2_S_4_O_6_) (Figure 4F). The results indicated that the addition of Na_2_S_2_O_3_ slightly reduced growth, yielding a final OD_600_ 0.70, lower than that of the H_2_-only control (0.79). In contrast, the addition of sodium tetrathionate significantly increased the exponential growth rate and resulted in a higher final biomass (OD_600_ 0.82) compared to the H_2_-only control. This suggests that ZZH C-3 could synergistically utilize both H_2_ and tetrathionate as energy sources for carbon fixation. This finding highlights a promising strategy for improving SCP production efficiency through multi-substrate co-utilization.

#### 3.4.5. Optimization of the Medium Composition by RSM

To systematically optimize the culture medium for biomass and protein production by *Thiomicrolovo* sp. ZZH C-3, this study employed a statistical optimization strategy integrating PB design, steepest ascent experiments, and RSM to accurately identify key influencing factors and their optimal concentrations.

##### PB Design for Screening Significant Factors

A preliminary PB design was used to screen 11 medium components, including macronutrients and micronutrients, in order to rapidly identify factors that significantly affect bacterial growth (Table S2). The model established from 15 experimental runs (including three center points) was highly significant (*p* < 0.0001), exhibited excellent goodness-of-fit (R^2^ = 0.9304), and demonstrated good predictive capability (predicted R^2^ = 0.8434), confirming the reliability of the model (Table S3). Analysis of ANOVA indicated that FeSO_4_·7H_2_O, CaCl_2_·2H_2_O, and MnSO_4_·H_2_O had *p*-values of <0.001, 0.0153, and 0.0209, respectively (all <0.05), which were identified as significant factors influencing the growth of ZZH C-3. Among them, FeSO_4_·7H_2_O showed the highest F-value (117.66), indicating its most prominent contribution to biomass accumulation. The result aligns with the essential physiological role of Fe^2+^ in HOB, wherein it functions as a key component of enzyme cofactors such as hydrogenase, ferritin, and cytochromes, directly participating in H_2_ oxidation, electron transport chain, and energy metabolism [44]. These processes provide the energy required for carbon fixation via the rTCA cycle.

##### Steepest Ascent Experiment to Approach the Optimal Response Region

Based on the PB experimental results, a steepest ascent experiment (Table S4) was conducted using the concentration variation in FeSO_4_·7H_2_O as the unit step size. The concentrations of CaCl_2_·2H_2_O and MnSO_4_·H_2_O were simultaneously adjusted according to the design coding formula to rapidly approach the region of maximum biomass response. The experiment revealed that the maximum biomass (OD_600_ 0.93) was achieved when the concentrations of FeSO_4_·7H_2_O, CaCl_2_·2H_2_O, and MnSO_4_·H_2_O were 9 mg/L, 0.16 g/L, and 2 mg/L, respectively. This point was therefore selected as the center point for the subsequent, more precise BBD of RSM.

##### Optimization Results by RSM

Based on the optimal region identified through the steepest ascent experiment, a three-factor, three-level BBD was implemented for response surface analysis (Table S6). The derived quadratic polynomial regression model is expressed as follows:

Y = 0.9596 + 0.0183A + 0.0141B − 0.0029C + 0.0075AB − 0.0115AC − 0.0017BC − 0.0357A^2^ − 0.0164B^2^ − 0.0239C^2^

(where Y represents the predicted OD_600_ value, and A, B, and C correspond to the coded values of FeSO_4_·7H_2_O, CaCl_2_·2H_2_O, and MnSO_4_·H_2_O, respectively).

Analysis of variance for the model (Table 3) demonstrated high significance (*p* < 0.0001), along with an excellent fit (R^2^ = 0.9908) and strong predictive accuracy (predicted R^2^ = 0.8929). The lack of fit was not significant (*p* = 0.1495 > 0.05), indicating that the model adequately represents the actual experimental data without substantial interference from unknown confounding factors. Both the linear and quadratic terms for FeSO_4_·7H_2_O (A) and CaCl_2_·2H_2_O (B) were highly significant (*p* < 0.0001), confirming their roles as key influencing factors. The parabolic relationship observed suggests the existence of optimal concentrations for maximizing biomass response. Although the linear term of MnSO_4_·H_2_O (C) was not significant (*p* = 0.1097), its quadratic term was highly significant (*p* < 0.0001), indicating a nonlinear influence of Mn^2+^ concentration on growth and underscoring the importance of maintaining an appropriate concentration.

**Figure 5:**
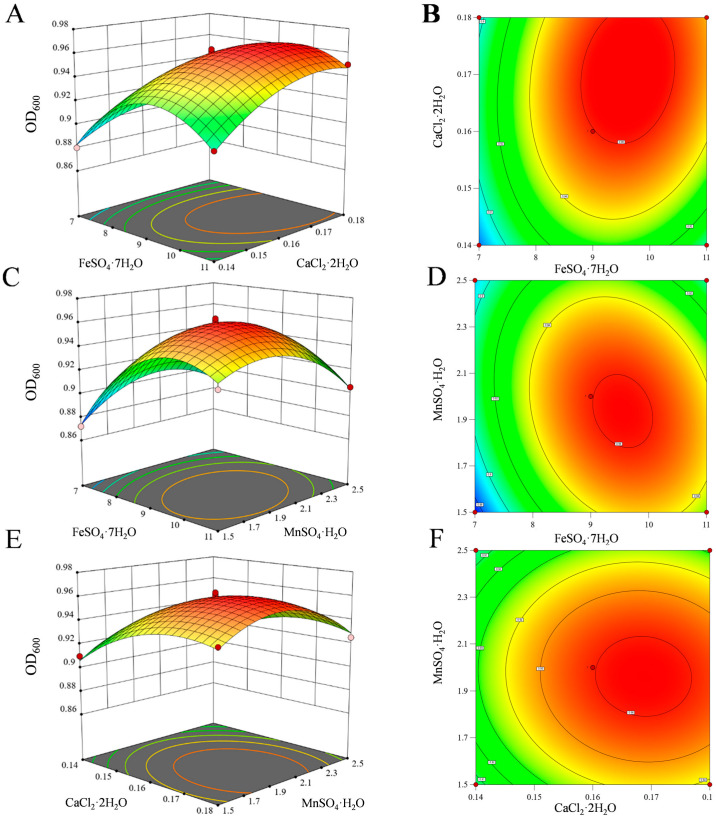
Interaction response of FeSO_4_·7H_2_O, CaCl_2_·2H_2_O, and MnSO_4_·H_2_O on Biomass yield. (**A**,**B**) Interaction between Fe^2+^ and Ca^2+^ (**C**,**D**) Interaction between Fe^2+^ and Mn^2+^ (**E**,**F**) Interaction between Ca^2+^ and Mn^2+^.

Response surface plots (Figure 5) and model term analysis collectively revealed complex interactions among the factors. The interaction between Fe^2+^ and Ca^2+^ was significant (*p* = 0.0118), as evidenced by the steepest slope of the response surface and the densest contour intervals, suggesting a synergistic relationship (Figure 5A,B). Fe^2+^ plays a central role in energy metabolism, whereas Ca^2+^ contributes to cell membrane stability, enzyme activity regulation, and signal transduction [45,46]. Together, they could interact with each other and collectively promote the growth of the strain ZZH C-3. In contrast, the interaction between Fe^2+^ and Mn^2+^ was highly significant (*p* = 0.0013) and exhibited a negative coefficient, indicating an antagonistic effect (Figure 5C,D). This may arise from excess Mn^2+^ competing for Fe^2+^ transmembrane transport sites or interfering with iron-sulfur cluster assembly, thereby impairing the activity of Fe^2+^-dependent enzymes [47]. The interaction between Ca^2+^ and Mn^2+^ was not significant (*p* = 0.4563), implying that their effects on bacterial growth operate independently (Figure 5E,F).

The model predicted that the maximum biomass (OD_600_ 0.9665) would be achieved with the following optimal medium composition: FeSO_4_·7H_2_O at 9.67 mg/L, CaCl_2_·2H_2_O at 0.17 g/L, and MnSO_4_·H_2_O at 1.92 mg/L.

##### Model Validation and Optimization Outcomes

To facilitate the conduct of experiments, the optimized conditions were adjusted to: FeSO_4_·7H_2_O 9.70 mg/L, CaCl_2_·2H_2_O 0.17 g/L, and MnSO_4_·H_2_O 1.90 mg/L. Triplicate validation experiments under these conditions produced an average OD_600_ of 0.95, which aligns closely with the predicted value (relative error ≈ 2.1%), thereby confirming the accuracy and reliability of the response surface model. Under optimized culture medium and conditions, the maximum biomass of strain ZZH C-3 reached 0.60 g/L, with a protein content of 73.56% and a protein yield of 0.44 g/L. This represents an approximate 14% increase in biomass compared to pre-optimization levels. These results clearly demonstrate that systematic medium optimization via statistical experimental design plays a crucial role in enhancing the production of SCP by Thiomicrolovo sp. ZZH C-3.

### 3.5. Growth Characteristics of ZZH C-3 Under Optimized Conditions

**Figure 6:**
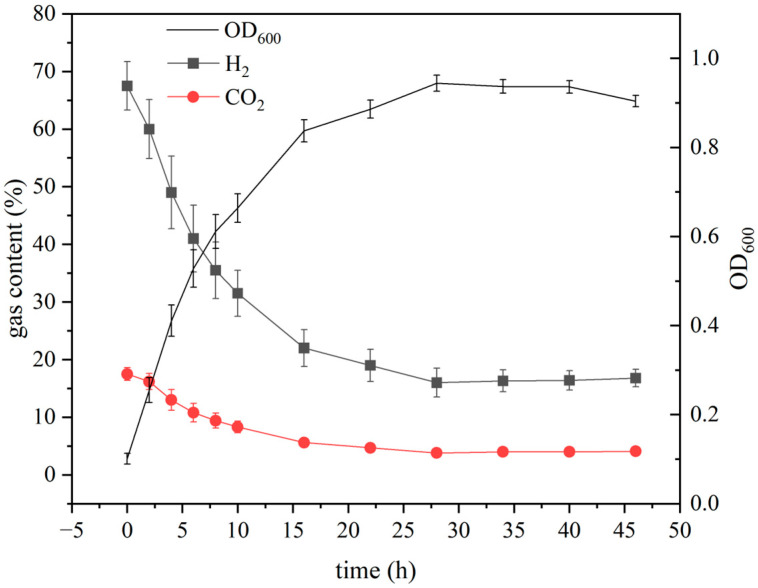
Growth curve and gas utilization of ZZH C-3.

Under optimized medium and culture conditions, the growth curve and gas consumption dynamics of strain ZZH C-3 were monitored by measuring the cell density (OD_600_) and headspace gas composition (Figure 6). The results indicated that ZZH C-3 entered the exponential growth phase immediately after inoculation without a detectable lag phase. The maximum specific growth rate (μ_max_) reached 0.46 h^−1^ during the initial phase (0–2 h), followed by a second exponential growth phase (2–4 h, μ = 0.24 h^−1^). The culture reached the stationary phase at 28 h, achieving a maximum OD_600_ of 0.94.

Quantitative analysis revealed an average molar consumption ratio of H_2_ to CO_2_ (H_2_/CO_2_) of 3.78, which is significantly lower than that typically observed in HOB dependent on the CBB cycle (e.g., *Cupriavidus necator*, with typical H_2_/CO_2_ ratios of 6.0–8.2) [48]. According to computational assessments in previous studies, the rTCA cycle harbored by ZZH C-3 is one of the least ATP-demanding pathways among all known natural carbon fixation routes. Its theoretical H_2_ consumption per molecule of CO_2_ fixed (2–4 mol H_2_/mol CO_2_) is substantially lower than that of the CBB cycle (4–6 mol H_2_/mol CO_2_) [13]. The low H_2_/CO_2_ ratio observed in ZZH C-3 provides direct evidence for the low energy cost characteristic of the rTCA pathway. Furthermore, ZZH C-3 achieved a biomass yield on hydrogen (Y_H2_) of 3.01 g/g H_2_, with a corresponding energy conversion efficiency from H_2_ to biomass (η _H2-bio_) as high as 42.5%, markedly exceeding that of traditional CBB pathway strains such as *C. necator* (typically 12–18%) (Table 4) [48,49]. Although gas consumption measurements in this study were based on headspace analysis, which may be influenced by pre-dissolved gases and air entrainment in the medium, the overall trend clearly underscores the superior substrate conversion and energy utilization efficiency of ZZH C-3. Although the current maximum biomass concentration (0.60 g DCW/L) remains lower than that of some industrial strains (e.g., *C. necator*), potentially due to gas–liquid mass transfer limitations at the laboratory scale, the high energy efficiency demonstrated here highlights considerable potential for large-scale, high-density cultivation.

### 3.6. Elemental Analysis and EPS Characterization of ZZH C-3

Elemental analysis indicated that the nitrogen content of ZZH C-3 cells was 11.77%, corresponding to a crude protein content of 73.56%. Based on the elemental composition, the elemental stoichiometry of the cells was determined to be C_1_H_1.83_O_0.45_N_0.23_. This ratio closely resembles the classic composition reported for HOB *C*. *necator* (C_1_H_1.85_O_0.40_N_0.22_) [41]. Beyond its protein content, strain ZZH C-3 exhibited valuable biotechnological traits during fermentation. It produced EPS at a yield of 0.07 g/L. EPS secretion, triggered during the late growth phase, facilitated auto-flocculation and rapid cell sedimentation, a trait that could substantially reduce downstream processing costs in industrial SCP production. Compositional analysis of the EPS by LC-MS revealed a complex heteropolysaccharide profile. Monosaccharides (e.g., xylose), disaccharides (e.g., sucrose, trehalose, turanose), the sugar alcohol mannitol, and the glycoside galactinol were identified among the hydrolysis products or associated metabolites (Table S7). Notably, the detection of N-acetylmuramic acid suggests the presence of peptidoglycan-derived fragments or glycosaminoglycan-like structures within the EPS matrix, which could enhance its structural stability and adhesive properties.

## 4. Conclusions

This study successfully isolated a marine HOB from mangrove sediment, named *Thiomicrolovo* sp. ZZH C-3, which can efficiently utilizes H_2_ as the energy source and CO_2_ as the carbon source for growth and SCP production via the reductive TCA cycle. Notably, compared with most microaerophilic rTCA-utilizing chemolithoautotrophs, this strainmaintained growth under oxygen levels up to 40%. After the optimization of fermentation conditions and medium composition, the maximum specific growth rate was 0.46 h^−1^, with a CDW of 0.60 g/L and reached a crude protein content of 73.56%. In addition, strain ZZH C-3 can produce 0.07 g/L of EPS, which promoted auto-flocculation, facilitating downstream separation. In summary, ZZH C-3 exhibits high oxygen resistance, high-efficiency carbon fixation capability, and self-flocculation ability, demonstrating strong potential for large-scale production in future. Future investigations should focus on process intensification to enhance cell density and growth rate.

## Figures and Tables

**Table 1:** Cellular fatty acid compositions (>1%) of strain ZZH C-3 and four type strains of the genus *Thiomicrolovo* Strains.

Fatty Acids (mol%)	1	2	3	4	5
C10:0	0.14	-	-	0.47	-
C12:0	1.25	0.49	0.55	0.58	0.4
Summed feature 2	0.29	-	-	0.53	1.02
13:0 iso 3OH	0.13	-	-	0.95	0.52
C14:0	12.53	10.43	12.77	10.94	10.83
C14:0 3-OH	4.77	3.71	5.48	3.62	3.3
Summed feature 3 (C16:1 ω7c/C16:1 ω6c)	33.55	36.9	40.8	32.92	33.09
C16:1 ω5c	0.38	0.57	0.72	0.22	0.2
C16:0	18.44	27.72	21.79	27.74	28.36
C18:0	0.38	0.23	1.28	1.58	0.42
Summed feature 8 (C18:1 ω7c/C18:1 ω6c)	13.98	19.23	15.31	16.67	20.84
C18:1ω5c	0.06	0.12	0.11	0.24	-
C18:1ω9c	0.22	-	0.23	-	0.36

Strains: 1, ZZH C-3; 2, *T. sulfuroxydans* HSL-3221; 3, *T. immobilis* HSL1-6; 4, *T. zhangzhouensis* HSL1-2; 5, *T. subterrani* HSL-1656 [31]. “-”, not detected (<0.1%).

**Table 2:** Comparison of key marker genes involved in the reductive tricarboxylic acid (rTCA) cycle and their copy numbers between *Thiomicrolovo* sp. ZZH C-3 and other strains within the genus.

Pathway	Enzyme	Short Name	ZZH C-3	HSL1–2	HSL-1656	HSL-3221	HSL1–6
rTCA cycle	ATP-dependent citrate lyase (ACL)	AclA	1	1	1	1	1
		AclB	1	1	1	1	1
	Oxoglutarate:ferredoxin oxireductase (OOR)	OorA	1	2	2	2	2
		OorB	2	2	2	2	2
		OorC	2	1	1	1	1
		OorD	1	1	1	1	1
		OorE	0	0	0	0	0
	Pyruvate:ferredoxin oxireductase (POR)	PorA	1	1	1	1	1
		PorB	1	1	1	1	1
		PorC	1	1	1	1	1
		PorD	1	1	1	1	1
		PorE	0	0	0	0	0

**Table 3:** Analysis of variance for the model regression.

Source	df	Sum of Squares	Mean Square	F-Value	*p*-Value	
Model	9	0.0149	0.0017	84.08	<0.0001	significant
A-FeSO_4_·7H_2_O	1	0.0027	0.0027	135.2	<0.0001	
B-CaCl_2_·2H_2_O	1	0.0016	0.0016	80.99	<0.0001	
C-MnSO_4_·H_2_O	1	0.0001	0.0001	3.36	0.1097	
AB	1	0.0002	0.0002	11.42	0.0118	
AC	1	0.0005	0.0005	26.84	0.0013	
BC	1	0	0	0.6216	0.4563	
A^2^	1	0.0054	0.0054	271.92	<0.0001	
B^2^	1	0.0011	0.0011	57.64	0.0001	
C^2^	1	0.0024	0.0024	122.3	<0.0001	
Residual	7	0.0001	0	-	-	
Lack of Fit	3	0.0001	0	3.13	0.1495	not significant
Pure Error	4	0	0	-	-	
Cor Total	16	0.0151	-	-	-	
R^2^ = 99.08%	Adj R^2^ = 97.9%					

**Table 4:** Comparison of cultivation and metabolic performance between the hydrogenotrophic strain ZZH C-3 and *Cupriavidus necator*.

Parameter	Unit	ZZH C-3	*Cupriavidus necator*
Maximum Biomass Concentration	g DCW/L	0.60	~1.448
Protein Content	% DCW	73.56	60~73
Maximum Specific Growth Rate	h^−1^	~0.46	~0.3
H_2_/CO_2_ Molar Consumption Ratio	mol H_2_/mol CO_2_	3.78	6.0~8.2
Energy Conversion Efficiency (η H_2_-bio)	%	42.5	12~18
Biomass Yield on H_2_ (YH_2_)	g DCW/g H_2_	3.01	1.1–1.4
Biomass Yield on CO_2_ (YCO_2_)	g DCW/g CO_2_	0.52	0.5~0.7

## Data Availability

The complete genome sequence of *Thiomicrolovo* sp. ZZH C-3 has been deposited in GenBank under the accession number JBTKXX000000000. The 16S rRNA gene sequence is available under accession number PX831749, and the hydrogenase large subunit sequences generated during this study are available under accession numbers PX778700–PX778703.

## Supplementary Materials

The following supporting information can be downloaded at: https://www.mdpi.com/article/10.3390/microorganisms14020346/s1, Table S1: Similarity analysis based on16S rRNA gene sequence of HOB isolated from mangrove sediments; Table S2: Plackett-Burman experimental design matrix for screening of important factors influencing the growth of ZZH C-3; Table S3: Statistical analysis of Plakette–Burman design; Table S4: Experimental designs and the results of steepest ascent; Table S5: Response surface test coefficients and levels; Table S6: The Box–Behnken experimental design with three independent variables; Table S7: Major components of EPS produced by strain ZZH C-3 identified by LC-MS analysis; Figure S1: Relationship between ZZH C-3 OD600 and CDW.
