# Supplementary material for: Isolation and Screening of Hydrogen-Oxidizing Bacteria from Mangrove Sediments for Efficient Single-Cell Protein Production Using CO2
Source: Microorganisms. 2026 Feb 2;14(2):346. doi: 10.3390/microorganisms14020346 (PMC12943458; doi:10.3390/microorganisms14020346)

Supplementary Materials for

Isolation and Screening of Hydrogen-Oxidizing Bacteria from Mangrove Sediments for Efficient Single-Cell Protein Production Using CO_2

Table S1 Similarity analysis based on16S rRNA gene sequence of HOB isolated from mangrove sediments.

Strain	Closest culturable species	Similarity (%)	Completeness (%)
ZZH A-4	*Sulfurovum sp.* XME15	98.78	98.1
ZZH B-6	*Marinobacterium sp.* AK27	99.08	97.0
ZZH C-3	*Thiomicrolovo sulfuroxydans* HSL-3221	99.67	100
ZZH D-4	*Sulfurovum indicum* ST-419(T)	95.14	100
ZZH D-6	*Sulfurimonas marina* B2(T)	88.82	89.7
ZZH F-3	*Sulfurimonas paralvinellae* G025	84.15	73.7
ZZH F-4	*Sulfurimonas indica* NW8N(T)	87.63	99.1
ZZH F-6	*Sulfurovum xiamenensis* XTW-4T	99.58	100

Table S2 Plackett-Burman experimental design matrix for screening of important factors influencing the growth of ZZH C-3.

Run	K_2HPO_4	$MgSO_4.7H_2O$	$CaCl_2.2H_2O$	$NiCl_2.6H_2O$	$Na_2SeO_3.5H_2O$	$ZnSO_4.7H_2O$	$FeSO_4.7H_2O$	$CuSO_4.5H_2O$	$Na_2MoO_4.2H_2O$	$MnSO_4.H_2O$	H_3BO_3	OD_{600}
	g/L	g/L	g/L	mg/L	mg/L	mg/L	mg/L	mg/L	mg/L	mg/L	mg/L	
1	0.15	1.2	0.15	0.5	1	5	5	0.1	0.1	1	0.5	0.9277
2	0.5	1.2	0.05	1	1	5	1	0.1	0.1	5	0.1	0.6826
3	0.5	0.2	0.15	1	0.5	5	5	0.5	0.1	1	0.1	0.8751
4	0.15	1.2	0.05	1	1	1	5	0.5	0.5	1	0.1	0.8715
5	0.325	0.7	0.1	0.75	0.75	3	3	0.3	0.3	3	0.3	0.7931
6	0.15	0.2	0.15	0.5	1	5	1	0.5	0.5	5	0.1	0.7423
7	0.15	1.2	0.15	1	0.5	1	1	0.5	0.1	5	0.5	0.7164
8	0.15	0.2	0.05	0.5	0.5	1	1	0.1	0.1	1	0.1	0.7664
9	0.5	0.2	0.05	0.5	1	1	5	0.5	0.1	5	0.5	0.8189
10	0.15	0.2	0.05	1	0.5	5	5	0.1	0.5	5	0.5	0.8296
11	0.5	1.2	0.15	0.5	0.5	1	5	0.1	0.5	5	0.1	0.892
12	0.5	1.2	0.05	0.5	0.5	5	1	0.5	0.5	1	0.5	0.7057
13	0.325	0.7	0.1	0.75	0.75	3	3	0.3	0.3	3	0.3	0.8136
14	0.5	0.2	0.15	1	1	1	1	0.1	0.5	1	0.5	0.7503
15	0.325	0.7	0.1	0.75	0.75	3	3	0.3	0.3	3	0.3	0.8207

Table S3 Statistical analysis of Plakette-Burman design

Source	Sum of Squares	df	Mean Square	F-value	p-value	
**Model**	0.0686	3	0.0229	44.56	< 0.0001	significant
C-$CaCl_2$:$2H_2O$	0.0044	1	0.0044	8.52	0.0153	
G-$FeSO_4.7H_2O$	0.0604	1	0.0604	117.66	< 0.0001	
K-$MnSO_4.H_2O$	0.0038	1	0.0038	7.5	0.0209	
Curvature	0.0003	1	0.0003	0.5576	0.4724	

Metric	Value
Std. Dev.	0.0227
R^2	0.9304
Mean	0.8004
Adjusted R^2	0.9095
C.V. %	2.83
Predicted R^2	0.8434
Adeq Precision	16.5048

Table S4 Experimental designs and the results of steepest ascent

Run	G-$FeSO_4.7H_2O$/mg/L	C-$CaCI_2.2H_2O$/g/L	K-$MnSO_4.H_2O$/mg/L	OD_{600}
1	3	0.1	3.5	0.80111
2	5	0.12	3	0.89113
3	7	0.14	2.5	0.87152
4	9	0.16	2	0.93034
5	11	0.18	1.5	0.88578
6	13	0.2	1	0.92232
7	15	0.22	0.5	0.92321
8	17	0.24	0	0.91698

Table S5 Response surface test coefficients and levels

Levels	$FeSO_4.7H_2O$(A) mg/L	$CaCl_2.2H_2O$(B) g/L	$MnSO_4.H_2O$(C) mg/L
− 1	7	0.14	1.5
0	9	0.16	2
1	11	0.18	2.5

Table S6 The Box-Behnken experimental design with three independent variables.

Run	A ($FeSO_4.7H_20$) (mg/L)	B ($CaCI_2.2H_20$) (g/L)	C ($MnSO_4.H_2O$) (mg/L)	OD_{600}
1	11	0.18	2	0.95
2	9	0.16	2	0.96
3	9	0.18	2.5	0.925
4	9	0.18	1.5	0.94
5	9	0.16	2	0.955
6	9	0.16	2	0.963
7	7	0.16	2.5	0.895
8	7	0.14	2	0.88
9	11	0.14	2	0.905
10	11	0.16	1.5	0.928
11	7	0.16	1.5	0.872
12	7	0.18	2	0.895
13	9	0.14	2.5	0.902
14	9	0.14	1.5	0.91
15	9	0.16	2	0.962
16	9	0.16	2	0.958
17	11	0.16	2.5	0.905

Table S7 Major components of (EPS produced by strain ZZH C-3 identified by LC-MS analysis

Compound	Type	Retention Time (min)	Molecular Formula	Ion Mode	Peak Intensity
Xylose	Monosaccharide	3.82	$C_5H_{10}O_5$	NEG	125.9
Ribose	Monosaccharide	3.69	$C_5H_{10}O_5$	NEG	23.2
Sucrose	Disaccharide	3.94	$C_{12}H_{22}O_{11}$	NEG	215.2
Trehalose	Disaccharide	3.93	$C_{12}H_{22}O_{11}$	NEG	215.2
Mannitol	Sugar alcohol	3.73	$C_6H_{14}O_6$	NEG	132.7
Galactinol	Glycoside	3.69	$C_{12}H_{22}O_{11}$	NEG	215.2
Turanose	Disaccharide	3.94	$C_{12}H_{22}O_{11}$	NEG	215.2
N-Acetylmuramic acid	Amino sugar derivative	3.69	$C_{11}H_{19}NO_8$	POS	154.2

Fig. S1 Relationship between ZZH C-3 OD_{600} and CDW

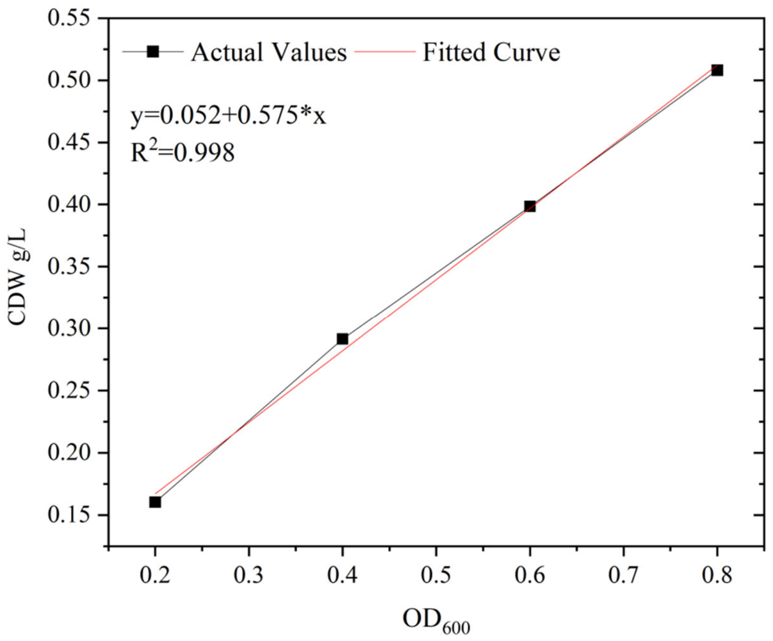

Supplement: Supplementary file 1 [file microorganisms-14-00346-s001.zip › microorganisms-4107710-supplementary.pdf]
